# Theory of the field-revealed Kitaev spin liquid

**DOI:** 10.1038/s41467-019-10405-8

**Published:** 2019-06-06

**Authors:** Jacob S. Gordon, Andrei Catuneanu, Erik S. Sørensen, Hae-Young Kee

**Affiliations:** 1 Department of Physics, University of Toronto, Toronto, ON M5S 1A7 Canada; 2 Department of Physics and Astronomy, McMaster University, Hamilton, ON L8S 4M1 Canada; 3 Canadian Institute for Advanced Research, CIFAR Program in Quantum Materials, Toronto, ON M5G 1M1 Canada

**Keywords:** Magnetic properties and materials, Phase transitions and critical phenomena, Topological matter

## Abstract

Elementary excitations in entangled states such as quantum spin liquids may exhibit exotic statistics different from those obeyed by fundamental bosons and fermions. Non-Abelian anyons exist in a Kitaev spin liquid—the ground state of an exactly solvable model. A smoking-gun signature of these excitations, namely a half-integer quantized thermal Hall conductivity, was recently reported in *α*-RuCl_3_. While fascinating, a microscopic theory for this phenomenon remains elusive because the pure Kitaev model cannot display this effect in an intermediate magnetic field. Here we present a microscopic theory of the Kitaev spin liquid emerging between the low- and high-field states. Essential to this result is an antiferromagnetic off-diagonal symmetric interaction which allows the Kitaev spin liquid to protrude from the ferromagnetic Kitaev limit under a magnetic field. This generic model displays a strong field anisotropy, and we predict a wide spin liquid regime when the field is perpendicular to the honeycomb plane.

## Abstract (web summary)

α-RuCl_3_ is a candidate material for studying Kitaev physics but a microscopic theory consistent with experiments has not yet been established. Here the authors present a theory with an intermediate-field spin liquid phase that can explain recent results indicating anyonic behaviour.

## Introduction

The Kitaev spin liquid (KSL) is a long-range entangled state on a honeycomb lattice^1^, which hosts non-Abelian^1–3^ anyon excitations in a magnetic field. It has been proposed that topological quantum computation can be performed via braiding of non-Abelian anyons^4^, meaning the KSL is of both practical, and fundamental interest. However, it has been challenging to find a solid state realization of Kitaev physics, which has been the focus of recent research. Several honeycomb materials have been suggested as KSL candidates, namely Mott insulators with strong spin-orbit coupling (SOC) featuring 4*d* or 5*d* transition metal elements^5–9^. Proposals so far include the iridates A_2_IrO_3_^5,6,10–13^ (A=Li, Na), and *α*-RuCl_3_^14–18^. However, all these candidates exhibit magnetic ordering at low temperatures^17,19–26^ which masks potential Kitaev physics. Later theoretical^27–29^ and experimental^30–33^ results suggest that *α*-RuCl_3_ may enter a field-induced spin liquid, but there has been no evidence that it is a chiral spin liquid until a half-integer quantized thermal Hall conductivity was reported in *α*-RuCl_3_^34^; a strong indication^35–37^ of chiral edge currents of Majorana fermions (MFs) predicted in a KSL.

While the observation of a half-integer quantized thermal Hall conductivity is the first experimental evidence of charge-neutral non-Abelian anyons in spin systems, a microscopic theory describing  their appearance under a field in *α*-RuCl_3_ is missing. This is because, if the dominant interaction in *α*-RuCl_3_ is the ferromagnetic (FM) Kitaev term (as shown through ab-initio studies^25,26^ and spin wave analysis^36^), the FM Kitaev phase is almost immediately destroyed, and the polarized state appears in an applied field^38–40^ with no intervening phase. This can be contrasted with the antiferromagnetic (AFM) Kitaev model which hosts a potentially gapless spin liquid under a field, supported by several numerical studies^39–47^. However, this intermediate gapless spin liquid cannot explain the half-integer thermal Hall effect observed in *α*-RuCl_3_. Thus, searching for a chiral spin liquid offering a half-integer thermal Hall effect in an intermediate magnetic field remains a challenging task.

Here we present a microscopic theory in which the KSL is revealed under a magnetic field. The key to our result is an AFM symmetric off-diagonal Γ interaction, which is essential to stabilize the otherwise fragile KSL under intermediate fields. The intermediate phase emerges between the low-field and high-field phases as Γ increases, and is adiabatically connected to the pure FM Kitaev phase at zero field, providing evidence that it is the KSL. We introduce a microscopic theory with a brief review of the generic nearest neighbor spin model for spin-orbit coupled honeycomb materials, appropriate for *α*-RuCl_3_.

## Results

### Model

**Fig. 1:**
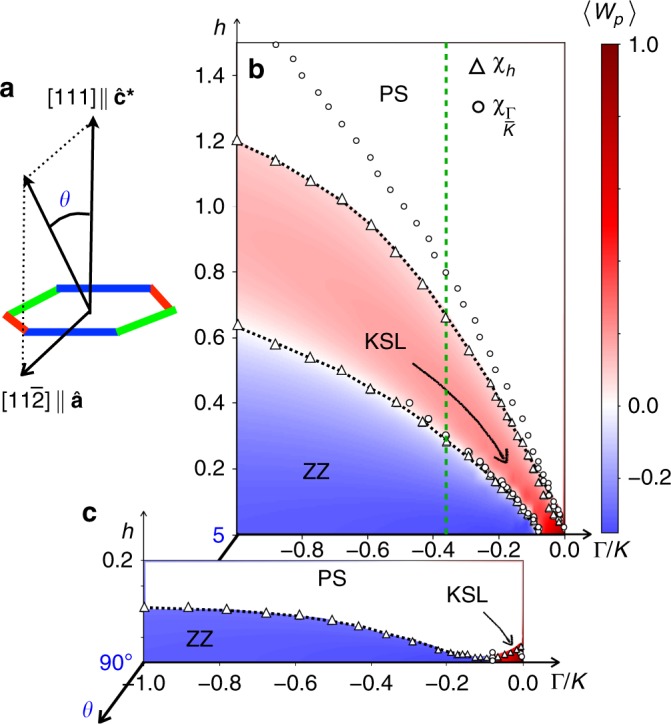
ED phase diagrams. **a** The angle *θ* is measured from [111] towards the in-plane $[11\bar 2]$ direction. Phase diagrams in the Γ/*K* − *h* plane (FM Kitaev and AFM Γ) obtained using ED on the 24-site honeycomb cluster are shown for **b** *θ* = 5° and **c** *θ* = 90° at a fixed Γ′ = −0.03 in units of $\sqrt {K^2 + {\mathrm{\Gamma }}^2} \equiv 1$. Circular and triangular markers represent peaks in the susceptibilities *χ*_Γ/*K*_ and *χ*_*h*_, respectively. The intermediate-field KSL is adiabatically connected to the pure *K* limit at *h* = 0, as indicated by a black arrow inside the KSL. Colors represent the expectation value of the plaquette operator 〈*W*_*p*_〉 in the ZZ and KSL, but not in the PS for clarity, which is discussed further in the main text. A sequence of phase transitions from ZZ order to the KSL, and finally the PS is found for *θ* = 5°, except near the pure *K* limit. The green line in **b** at Γ/*K* ≃ −0.37 denotes a representative slice where *χ*_*h*_ and *S*(**q**) are plotted in Fig. 2

The nearest neighbor model has been derived in refs. ^5,12,13^ based on a strong coupling expansion of the Kanamori Hamiltonian. The combination of crystal field splitting and strong spin-orbit coupling leads to a model based on pseudospin-${\textstyle{1 \over 2}}$ local moments with bond-dependent interactions. On a bond of type *γ* ∈ {*x*, *y*, *z*} with sites *j*, *k*, the nearest-neighbor spin Hamiltonian is taken to be of the *J*-*K*-Γ-Γ′ form^13^

1 $$\begin{array}{*{20}{l}} {{\cal{H}}_{jk}^\gamma } \hfill & { = J{\mathbf{S}}_j \cdot {\mathbf{S}}_k + KS_j^\gamma S_k^\gamma + {\mathrm{\Gamma }}\left( {S_j^\alpha S_k^\beta + S_j^\beta S_k^\alpha } \right)} \hfill \\ {} \hfill & \,\,\,\,{ + \,\,{\mathrm{\Gamma }}^{\prime} \left( {S_j^\alpha S_k^\gamma + S_j^\gamma S_k^\alpha + S_j^\beta S_k^\gamma + S_j^\gamma S_k^\beta } \right)} \hfill \end{array},$$

where *α*, *β* are the remaining spin components in {*x*, *y*, *z*}/{*γ*}. The spin components are directed along the cubic axes of the underlying ligand octahedra, so the honeycomb layer lies in a plane perpendicular to the [111] spin direction as shown in Fig. 1a. A small Γ′ is present due to trigonal distortion of ligand octahedra in the real material. Here we omit the Heisenberg *J* for simplicity, and its effects are discussed later. Earlier studies^14,26,29,48,49^ noted that the Γ interaction with AFM sign may play an important role near the FM Kitaev regime to stabilize the spin liquid^48^. Since *α*-RuCl_3_ has a dominant FM Kitaev interaction with AFM Γ, we focus on Γ/*K* ∈ [−1, 0] with Γ > 0 and *K* < 0. The remaining parameters of the Hamiltonian are expressed in units of $\sqrt {K^2 + {\mathrm{\Gamma }}^2} \equiv 1$.

To describe the effect of a magnetic field we consider a Zeeman term with isotropic *g*-factor

2 $${\cal{H}}_Z = - h\mathop {\sum}\limits_j {{\widehat{\mathbf{h}}} \cdot {\mathbf{S}}_j} ,$$

where *h* is the magnetic field strength, and ${\widehat{\mathbf{h}}}$ is a unit vector specifying the field direction. The effect of an anisotropic *g*-factor is discussed later. In order to make a connection with the thermal Hall measurements^34^ we focus on field directions in the ${\hat{\mathbf{a}}}{\hat{\mathbf{c}}}^ \ast$ plane spanned by $[11\bar 2]$ and [111]. The direction of the field in this plane is parameterized by an angle *θ* from the [111] direction, as shown in Fig. 1a.

### Exact diagonalization

Our main results are shown in Fig. 1b, c. Phase diagrams in the Γ/*K* − *h* plane are shown for tilting angles (b) *θ* = 5° and (c) 90° obtained through numerical exact diagonalization (ED) with fixed Γ′ = −0.03 and *J* = 0. Details of the 24-site honeycomb cluster used are discussed in Supplementary Note 1. Peaks in the susceptibilities $\chi _{{\mathrm{\Gamma }}/K} = - \partial _{{\mathrm{\Gamma }}/K}^2e_0$ and $\chi _h = - \partial _h^2e_0$, where *e*_0_ = *E*_0_/*N* is the ground state energy density, are depicted as circles and triangles, respectively. There are three phases in the phase space, namely, zigzag (ZZ) magnetic order at low fields, the KSL, and a polarized state (PS) at high fields. Remarkably, we find an intermediate KSL sitting between ZZ order and the PS which is adiabatically connected to the pure *K* limit at *h* = 0.

The intermediate KSL begins from the pure FM *K* regime, i.e., bottom right corner of the phase diagram, which is unstable to a small magnetic field. However, it is stabilized by the AFM Γ term and extends above the ZZ phase in a magnetic field. For moderate Γ/*K* appropriate for *α*-RuCl_3_—for example, Γ/*K* ≃ −0.37 indicated by the green dashed line in Fig. 1b—we observe two phase transitions from ZZ order to the KSL, and then from KSL to the PS as the field increases. Close to the Kitaev limit, the peak positions of the magnetic (*χ*_*h*_) and Γ/*K* (*χ*_Γ/*K*_) susceptibilities agree well along the phase boundaries between the KSL and PS for each direction. At larger Γ/|*K*| the singular points determining the phase boundaries start to deviate for *θ* = 5°, as seen through differing positions of circular and triangular markers in Fig. 1b. However, the anomalous peaks in *χ*_Γ/*K*_ in this region shrink significantly while the peaks in *χ*_*h*_ retain their sharpness. Since these peaks are not seen in *χ*_*h*_, and there are strong variations within the PS in the quantities discussed below, we determine the KSL-PS phase boundary based on peaks in *χ*_*h*_ and attribute peaks in *χ*_Γ/*K*_ to large fluctuations above the KSL.

Interestingly, with a constant Γ′ both the ZZ and KSL phases widen with increasing Γ as suggested by the curvature of the transition line in Fig. 1b. This behavior survives for further tilting of the magnetic field away from [111] with increased $[11\bar 2]$ in-plane component. However, the window of the KSL rapidly diminishes with tilting angle until a direct transition between ZZ order and the PS appears at large Γ/|*K*|, as shown in Fig. 1c for a $[11\bar 2]$ field. The critical field required to destroy the ZZ ordering drops dramatically with increasing *θ*. With an estimate of the energy unit as $\sqrt {K^2 + {\mathrm{\Gamma }}^2} \sim 7\,{\mathrm{meV}}$, *h* = 0.1 corresponds to a field of ~10 T. This is within the range of fields required to kill the ZZ order in *α*-RuCl_3_^34^.

Since the pure Kitaev limit at *h* = 0 involves the fractionalization of spins into itinerant MFs and ${\Bbb Z}_2$ fluxes, another quantity that characterizes the KSL is the plaquette operator *W*_*p*_^1^

3 $$W_p = 2^6S_1^xS_2^yS_3^zS_4^xS_5^yS_6^z,$$

defined on sites belonging to a hexagonal plaquette *p*. The pure KSL with *h* = Γ = Γ′ = 0 (bottom right corner of the phase diagram) is a flux-free state with *W*_*p*_ = +1 on all plaquettes. A finite Γ, Γ′ or *h* spoils the exact solubility of the Kitaev model, as they generate interactions among the MFs and ${\Bbb Z}_2$ fluxes. Despite the fact that plaquette operators are no longer conserved quantities 〈*W*_*p*_〉 remains positive in the KSL, denoted by red colors in Fig. 1. At the same time the plaquette expectation value is negative in the ZZ ordered phase, as denoted by blue colors in Fig. 1, distinguishing it from the neighboring KSL. Due to this sign difference, the phase boundary between KSL and ZZ is accompanied by a vanishing 〈*W*_*p*_〉 and seen through the rapid color change in Fig. 1. This is a generic feature which also appears for an in-plane field of *θ* = 90°. Further details of the negative plaquette expectation in the ZZ phase can be found in Supplementary Note 3.

**Fig. 2:**
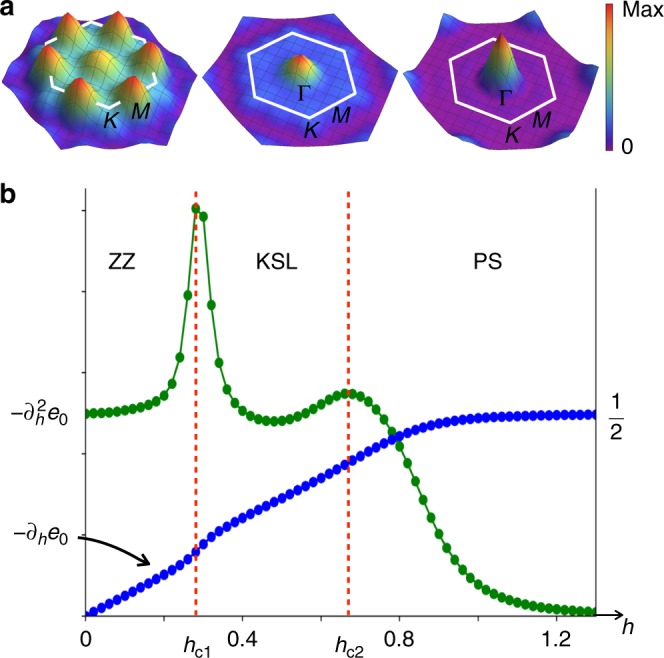
Sequence of phases in a magnetic field. **a** Spin structure factor *S*(**q**) within the ZZ, KSL, and PS phases. **b** $\chi _h = - \partial _h^2e_0$ and magnetization *m* = −∂_*h*_*e*_0_ as a function of a 5° tilted field are shown for a fixed Γ/*K* ≃ −0.37, indicated by a green dashed line in Fig. 1b. The field strengths *h*_c1_ and *h*_c2_ are the critical values at which the transitions from the ZZ to KSL, and the KSL to PS occur, respectively

To confirm the ZZ magnetically ordered phase at low field, and the sequence of phase transitions, we compute the spin structure factor *S*(**q**) for increasing values of *h* along the green dashed line of Fig. 1b where Γ/*K* ≃ −0.37. For low fields of *h* < *h*_c1_, where *h*_c1_ is the position of the first transition, *S*(**q**) displays sharp features at the **M**-point of the Brillouin zone (BZ) consistent with ZZ magnetic order as shown in Fig. 2a. Within the intermediate phase, *h*_c1_ < *h* < *h*_c2_ where *h*_c2_ is the position of the second transition, *S*(**q**) is diffuse with a soft peak at the **Γ**-point. Interestingly, an increased intensity at the **Γ**-point was observed in *α*-RuCl_3_ under fields in a recent neutron scattering measurement^50^. As expected, the PS exhibits a sharp feature at the **Γ**-point for *h* > *h*_c2_. Note that the magnetization *m* = −∂_*h*_*e*_0_ eventually saturates at ${\textstyle{1 \over 2}}$ in the PS at a field larger than *h*_c2_, as shown in Fig. 2b indicating large spin fluctuations inside the PS.

### Competition between ZZ and KSL

ZZ ordering at low fields can be traced back to the presence of other small interactions, such as such as a FM Γ′^5,12,13,27^ and/or FM (*J*) and AFM third-nearest neighbor (*J*_3_) Heisenberg interactions^51^. We have considered Γ′ as a minimal nearest-neighbor perturbation away from the *K* − Γ model which induces ZZ order at low fields. The combination of *J* < 0 and *J*_3_ > 0 has a similar effect to Γ′ in that their combination can also induce ZZ ordering^13,51^. Inclusion of these small terms would not alter our main results as they further stabilize the ZZ phase. However, the combined strength of terms stabilizing the ZZ order should be small enough to maintain the intermediate KSL.

**Fig. 3:**
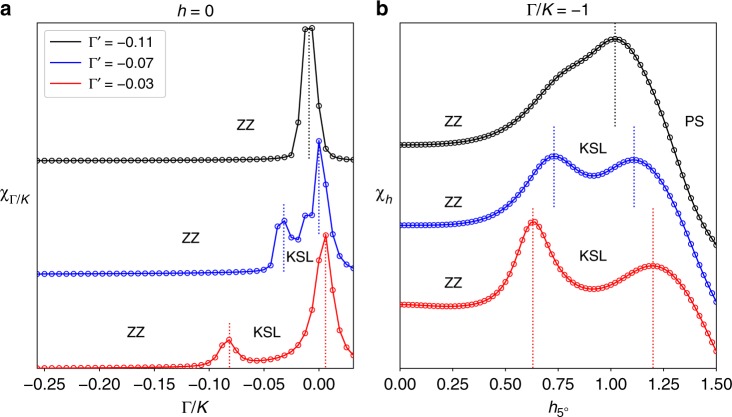
Effect of Γ′ on the intermediate phase. The role of Γ′ is illustrated through cuts of the susceptibilities for increasing values of |Γ′| as calculated with ED on a 24-site honeycomb cluster. **a** The susceptibility *χ*_Γ/*K*_ is shown at *h* = 0 near the Kitaev limit for Γ′ = −0.03, −0.07, and −0.11. Peak values of *χ*_Γ/*K*_ are scaled to the same number and are offset for visibility. With increasing |Γ′| the ZZ phase is seen to expand as the KSL shrinks, and the KSL disappears entirely beyond ${\mathrm{\Gamma }}_{\mathrm{c}}^\prime \simeq - 0.1$. **b** The magnetic susceptibility *χ*_*h*_ is shown for a 5° tilted field at Γ/*K* = −1 where the intermediate phase is largest for Γ′ = −0.03. Values of *χ*_*h*_ are not scaled, but simply offset for visibility. A similar trend is found where the size of the intermediate phase shrinks with increasing |Γ′|. The intermediate KSL disappears beyond ${\mathrm{\Gamma }}_{\mathrm{c}}^\prime \simeq - 0.1$ where the KSL at *h* = 0 is overtaken by ZZ magnetic order. Vertical dashed lines represent the position of phase transitions

The particular values of Γ′ used in our calculations were chosen based on the following criteria. For Γ′ = 0 the phase to the left of the KSL at larger Γ/|*K*| is non-magnetic, as discussed in the Underlying Phase Diagram section, and develops ZZ magnetic order as Γ′ < 0 is introduced. The magnitude |Γ′| was chosen to be the smallest value such that ZZ magnetic order develops within this phase. If |Γ′| becomes too large then the KSL at *h* = 0 will be wiped out entirely. To quantify this, we have calculated *χ*_Γ/*K*_ with ED on a 24-site honeycomb cluster near the Kitaev limit at *h* = 0 for different values of Γ′. As |Γ′| increases, the ZZ ordered phase expands while the KSL shrinks at *h* = 0 as shown in Fig. 3a. The KSL at *h* = 0 is found to disappear entirely beyond ${\mathrm{\Gamma }}_{\mathrm{c}}^\prime \simeq - 0.1$. Evolution of the intermediate-field KSL with Γ′ is seen in Fig. 3b through the magnetic susceptibility *χ*_*h*_ in a 5° field at Γ/*K* = −1, where the intermediate phase is largest with Γ′ = −0.03. Emergence of the intermediate-field KSL depends crucially on the survival of the pure Kitaev phase at *h* = 0, because *χ*_*h*_ shows a single transition from ZZ to PS beyond ${\mathrm{\Gamma }}_{\mathrm{c}}^\prime \simeq - 0.1$. Quantifying the strengths of Γ′, *J*, *J*_3_ required for an intermediate KSL is left for a detailed future study.

### DMRG

In order to check the dependence of this result on cluster geometry, we have also studied a two-leg honeycomb strip using density-matrix renormalization group (DMRG). We denote the total number of sites in the strip by *N*. This geometry has recently been used to study the Kitaev-Heisenberg model^52^, where it was found that its phase diagram displays a striking similarity with that of the 2D honeycomb lattice. For the *K* − Γ model we find quantitative differences in the positions of the phase boundaries due to the cluster connectivity, but the main result of an emerging intermediate-field KSL remains the same. Further rationale for this choice of geometry is discussed in Supplementary Note 1.

**Fig. 4:**
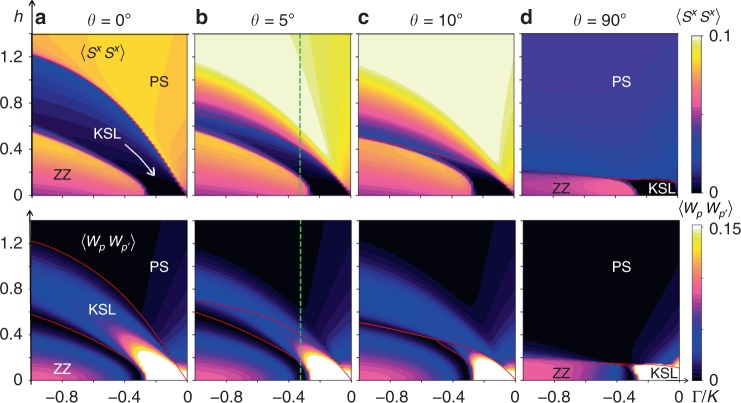
DMRG phase diagrams. The spin–spin correlator $\left\langle {S_j^xS_k^x} \right\rangle$ at *k* − *j* = 50 along the leg of the strip with maximal correlations is shown in the first row, and the plaquette-plaquette correlator $\left\langle {W_pW_{p\prime }} \right\rangle$ at *p*′ − *p* = 30 in the second as a function of Γ/*K* and *h* with Γ′ = −0.1. These are obtained in the two-leg honeycomb strip using DMRG with *N* = 200 and OBC. The field directions are constant in a given column, which are **a** *θ* = 0°, **b** *θ* = 5°, **c** *θ* = 10°, and **d** *θ* = 90° ($\left[ {11\bar 2} \right]$ in-plane). Smooth curves fitted to peaks in either *χ*_*h*_, or *χ*_Γ/*K*_ are drawn with red lines. The white arrow in the spin–spin correlators of **a** indicates the smooth connection from the intermediate phase to the pure Kitaev limit. The dashed green line at Γ/*K* = −0.325 indicates a representative slice where certain quantities are plotted in Fig. 5 for a *θ* = 5° tilted field

Phase diagrams in the Γ/*K* − *h* plane with *N* = 200 and open boundary conditions (OBC) for tilting angles *θ* = 0°, 5°, 10°, and 90° with fixed Γ′ = −0.1 and *J* = 0 are shown in Fig. 4. The phase boundaries in Fig. 4, determined by peaks in *χ*_*h*_ or *χ*_Γ/*K*_, are represented by red lines. We find a qualitative similarity with the ED phase diagram of Fig. 1, showing a region of KSL which extends above the ZZ ordered phase and below the PS under a magnetic field. The KSL phase space shrinks rapidly as the in-plane component of the field becomes larger. As found with ED on the 24-site honeycomb cluster, the intermediate KSL at large Γ/|*K*| disappears as the field tilts towards *θ* = 90°, leaving a single direct transition from ZZ to the PS. Crucially, a small region of KSL remains intact at smaller Γ/|*K*|. Thus, with the in-plane $[11\bar 2]$ field the KSL is confined to a narrow range of low fields near the pure FM Kitaev limit. The same qualitative behavior is seen for another in-plane field direction of $[\bar 110]$ only for the 24-site honeycomb cluster used with ED. On the two-leg honeycomb strip the KSL (in the region Γ/|*K*| < Γ/|*K*|_c_ where Γ/|*K*|_c_ is the transition point between ZZ and KSL at *h* = 0) is immediately destroyed by any non-zero field in this direction, as shown in Supplementary Fig. 3. While this is consistent with the observation that there is no ${\Bbb Z}_2$ topological order when a certain mirror symmetry is preserved^46^, the phase boundary is an artifact of the strip geometry.

The first row in Fig. 4 shows $\left\langle {S_j^xS_k^x} \right\rangle$ at separation *k* − *j* = 50 along the leg of the strip with maximum correlations as a function of field and Γ/*K* for different tilting angles of the field in the ${\hat{\mathbf{a}}}{\hat{\mathbf{c}}}^ \ast$ plane. As expected, correlations are appreciable within the magnetically ordered and polarized states. The KSL phase is clearly distinguished from the surrounding ordered states by nearly vanishing $\left\langle {S^xS^x} \right\rangle$ ($= \left\langle {S^yS^y} \right\rangle$) spin correlations. However, spin-spin correlations need not be identically zero except at *h* = 0 due to a component of the spin aligning with the field, which is more pronounced when *h* is large. The PS away from the pure Kitaev region shows large spin fluctuations, which is similar to the 24-site ED result where saturation of the magnetization occurs at higher fields above *h*_c2_, and small peaks in *χ*_Γ/*K*_ only appear above the KSL-PS phase boundary.

In the bottom row of Fig. 4 we show plaquette-plaquette correlations $\left\langle {W_pW_{p\prime }} \right\rangle$ at separation *p*′ − *p* = 30. Close to the Kitaev limit these correlations are nearly unity, consistent with 〈*W*_*p*_〉 = +1 in the *K* limit, and decrease with increasing field and Γ/|*K*| within the KSL. This is expected because the magnetic field, as well as Γ, Γ′, introduces interactions among the MF and flux degrees of freedom. Interestingly, the plaquette–plaquette correlations, which approach $\left\langle {W_p} \right\rangle ^2$ in the KSL at large separations, show large fluctuations in the PS above the KSL phase for 5° and 10° tilting angles. We note that these fluctuations in the PS for *θ* = 5° and 10° disappear above the transition line between the KSL and PS determined by *θ* = 0°.

**Fig. 5:**
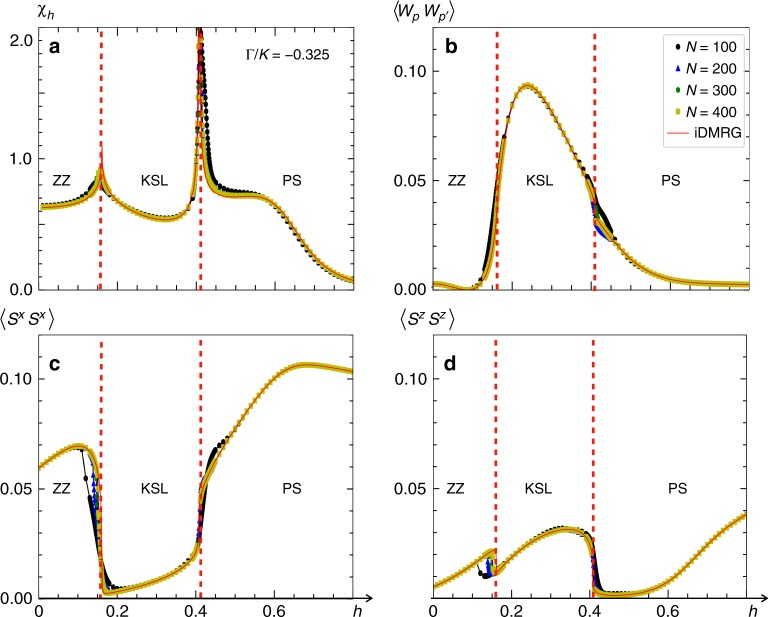
Evolving correlations in a magnetic field. With Γ/*K* = −0.325 and Γ′ = −0.1, quantities computed in the two-leg honeycomb strip with DMRG are plotted as a function of a 5° field for system sizes *N* = 100, 200, 300, 400 and infinite DMRG (iDMRG) as solid red line. **a** *χ*_*h*_ displays two peaks which sharpen with increasing system size. These transitions separate ZZ magnetic order and the PS from the intervening KSL. **b** Plaquette–plaquette correlations $\left\langle {W_pW_{p\prime }} \right\rangle$ are small within the ZZ and polarized phases, and become appreciable within the KSL. **c** Conversely, the *xx* spin–spin correlations 〈*S*^*x*^*S*^*x*^〉 are small within the KSL and large in the magnetically ordered phases. **d** Sharp changes in the *zz* spin–spin correlations are seen across the phase boundaries, and display a finite value consistent with a KSL in field. Spin and plaquette correlations are evaluated at the largest separation accessible in each cluster

A representative cut of the phase diagram is presented in Fig. 5c, d as a function of a 5° tilted field with Γ/*K* = −0.325, which corresponds to the green line in Fig. 4b. With increasing field, a sequence of transitions from ZZ order to the KSL and finally the PS are evidenced by strong singular behavior in *χ*_*h*_ in Fig. 5a. The transition between ZZ order and the KSL is accompanied by a sharp increase in plaquette–plaquette correlations shown in Fig. 5b, and a larger value of 〈*W*_*p*_〉 accordingly. Note that the maximum value of $\left\langle {W_pW_{p\prime }} \right\rangle \simeq 0.1$ corresponds to 〈*W*_*p*_〉 ≃ 0.32, as $\left\langle {W_pW_{p\prime }} \right\rangle$ approaches $\left\langle {W_p} \right\rangle ^2$ at large distances. Components $\left\langle {S^xS^x} \right\rangle$ and $\left\langle {S^zS^z} \right\rangle$ of the spin–spin correlators are plotted in Fig. 5c, d. While the $\left\langle {S^xS^x} \right\rangle$ correlations are small in the KSL, the $\left\langle {S^zS^z} \right\rangle$ correlations are slightly larger. This is similar to the honeycomb cluster with ED, where a finite magnetization *m* is present in the KSL phase, as shown in Fig. 2b. The source of asymmetry between the *S*^*x*^ and *S*^*z*^ components of the spin is two-fold. One is due to the two-leg honeycomb strip connectivity, and the second is the finite tilting of the magnetic field which further enhances their difference. The preceding properties are shown for *N* = 100, 200, 300, 400, and iDMRG in Fig. 5 with different colors, and are seen to be relatively insensitive to the system size.

### Underlying phase diagram

**Fig. 6:**
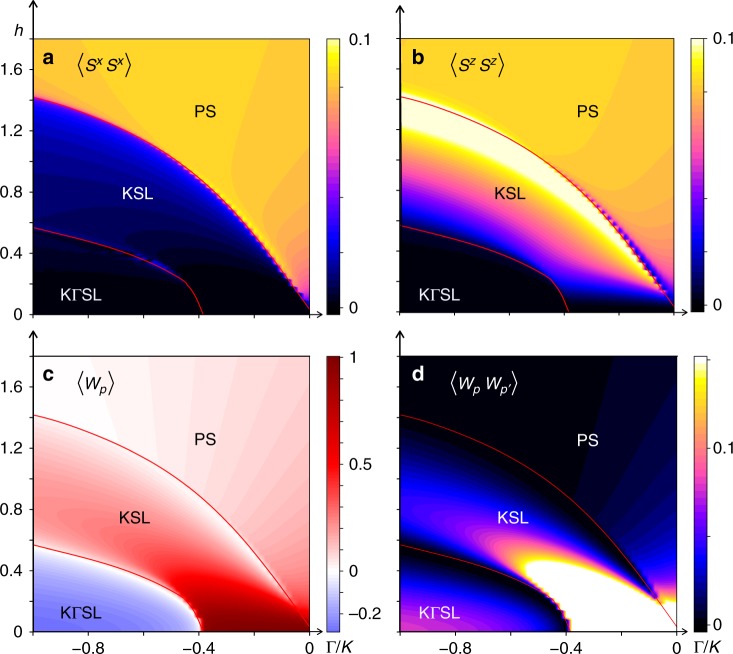
Underlying phase diagram. Underlying phase diagram with Γ′ = 0 and *θ* = 0 in the Γ/*K* − *h* plane as calculated with DRMG for *N* = 200 and OBC. Spin–spin correlations **a** $\left\langle {S_j^xS_k^x} \right\rangle$ and **b** $\left\langle {S_j^zS_k^z} \right\rangle$ at *k* − *j* = 50 are absent within the KΓSL and the KSL phases at *h* = 0, and develop under the field. Phase boundaries, determined by smoothed fits to the peaks of of *χ*_*h*_ and *χ*_Γ/*K*_, are drawn as red lines, and separate the KΓSL and the PS from the intervening KSL. **c** Plaquette expectation 〈*W*_*p*_〉 differentiates the KΓSL and the KSL as it changes sign across the transition. **d** Plaquette–plaquette correlations $\left\langle {W_pW_{p\prime }} \right\rangle$ at *p*′ − *p* = 30 are small in the PS and become appreciable within the KΓSL and KSL, approaching $\left\langle {W_p} \right\rangle ^2$ in the limit of large separation

To understand the microscopic mechanism of the emerging KSL, we study the *K* − Γ model without Γ′—i.e., in the absence of small interactions that induce ZZ order—at *θ* = 0. At zero field, there is a finite region of the KSL when the AFM off-diagonal symmetric Γ interaction is introduced. Then in zero field at Γ/*K*_c_ there is a transition from the KSL to another possible spin liquid dubbed KΓ spin liquid (KΓSL)^28^. Components of the spin-spin correlators, $\left\langle {S^xS^x} \right\rangle$ and $\left\langle {S^zS^z} \right\rangle$ shown in Fig. 6a, b, demonstrate a lack of magnetic order in the KSL and KΓSL at *h* = 0, and finite correlations building with increasing field.

The KΓSL is characterized by a finite $\langle {W_pW_{p\prime }} \rangle$ like the KSL, but with negative 〈*W*_*p*_〉 as shown in Fig. 6c, d. While 〈*W*_*p*_〉 is positive in the KSL, a negative 〈*W*_*p*_〉 in the KΓSL indicates a phase with a finite flux density. Strikingly, when the field is applied along the [111] direction the KSL sits above the KΓSL for a fixed Γ/*K*, leading to two phase transitions with increasing field: KΓSL → KSL → PS. The KΓSL is extremely fragile to additional interactions that stabilize ZZ order. For example, when a small Γ′ interaction is introduced the KΓSL is replaced by the ZZ ordered phase as shown in Fig. 1. Importantly, the ZZ order does not extend all the way to the Kitaev limit and leaves a finite region of the KSL at zero field.

## Discussion

Here we presented a microscopic theory, based on dominant Kitaev and off-diagonal symmetric Γ interactions, which offers an intermediate KSL emerging between the low-field and high-field states. The low-field ZZ state is induced via small perturbations beyond *K* and Γ, such as Γ′ due to trigonal distortion of ligand octahedra. Our numerical data indicates that this additional interaction can wipe out the KSL phase (including the pure Kitaev limit at *h* = 0) when its strength is too large, leading to a single transition between ZZ and PS under a magnetic field. Experimental reports of a half-quantized thermal Hall conductivity in *α*-RuCl_3_ imply that the strengths of interactions beyond *K* and Γ are small enough to leave the intermediate KSL intact, yet finite to induce the ZZ order. In the absence of these interactions the K-Γ model exhibits another possible spin liquid called the KΓSL, which is then replaced by the ZZ due to these small interactions. It is possible that a region of the KΓSL survives with smaller Γ′ while developing ZZ order, resulting in two spin liquids between ZZ and PS under a field. We leave these questions for a future study.

As the magnetic field is tilted away from the out-of-plane [111] direction towards the in-plane $[11\bar 2]$ direction the intermediate KSL region shrinks rapidly—independent of the strength of Γ′ and for both cluster shapes studied here. What remains is a small intermediate phase at fields an order of magnitude smaller for moderate Γ/|*K*|, showing a dramatic magnetic anisotropy. Considering an anisotropic *g*-factor, due to a combination of the layered structure and SOC, the magnetic anisotropy is further enhanced by the ratio between the in-plane *g*_ab_ and the out-of-plane $g_{{\mathrm{c}}^ \ast }$ components. While smaller tilting angles are less effective at destroying the ZZ magnetic order, they offer a much larger region of the KSL. To enlarge the intermediate KSL phase, we therefore propose that a field should be applied at smaller tilting angles. Further thermal Hall transport measurements for different in-plane components would be desirable in order to test our microscopic theory.

There are several aspects of this work that require further study. The first is the presence of large fluctuations in $\left\langle {W_pW_{p\prime }} \right\rangle$ and $\left\langle {S_j^xS_k^x} \right\rangle$ just above the KSL phase into the PS, which is also seen by *χ*_Γ/*K*_ and an unsaturated magnetization in the 24-site honeycomb cluster. This is suggestive of a non-trivial crossover region into the fully polarized phase. We also note the presence of a non-magnetic phase dubbed KΓSL in the underlying phase diagram of the *K*-Γ model on the two-leg geometry next to the KSL phase. The KΓSL at zero field is differentiated from the KSL by a sharp drop from 〈*W*_*p*_〉 = 1 in the KSL to $\left\langle {W_p} \right\rangle \simeq - \frac{1}{3}$ in the KΓSL, accompanied by a singular *χ*_Γ/*K*_. Nature of the KΓSL, numerical studies of this phase in the honeycomb geometry,  and the transition to the KSL are excellent subjects for future study. For instance, studies of possible vortex patterns due to strong interactions among MFs and ${\Bbb Z}_2$ vortices would be highly interesting to pursue.

## Methods

### Details of simulations

Numerical exact diagonalization (ED), and density matrix renormalization group (DMRG) are used to study the parameter space appropriate for *α*-RuCl_3_. In the ED and DMRG calculations, we consider the two-leg honeycomb strip geometry, and the 24-site honeycomb shape in ED only, both shown in Supplementary Fig. 1. The choice of these clusters is discussed in Supplementary Note 1, and is related to hidden points of SU(2) symmetry present in the 2D limit.

ED was performed on the 24-site honeycomb cluster with periodic boundary conditions, where the Lanczos method was used to obtain the lowest-lying eigenvalues and eigenvectors of the Hamiltonian in Eq. (1).

Part of the numerical calculations were performed using the ITensor library (http://itensor.orghttp://itensor.org) typically with a target precision of 10^−11^ using up to 1000 states. All DMRG calculations were performed using open boundary conditions (OBC). The iDMRG calculations were performed using a target precision of 5 × 10^−11^ and up to 1000 states.

## Supplementary information

Supplementary Information

## Data Availability

The data that support the findings of this study are available from the corresponding author upon reasonable request.

## Code availability

The code used to generate the data used in this study is available from the corresponding author upon reasonable request.
