# Supplementary material for: Theory of the field-revealed Kitaev spin liquid
Source: Nat Commun. 2019 Jun 6;10:2470. doi: 10.1038/s41467-019-10405-8 (PMC6554414; doi:10.1038/s41467-019-10405-8)
Supplement: Supplementary file 1 — Supplementary Information [file 41467_2019_10405_MOESM1_ESM.pdf]

Supplementary Information:
Theory of the field-revealed Kitaev spin liquid

Jacob S. Gordon,[1] Andrei Catuneanu,[1] Erik S. Sørensen,[2] and Hae-Young Kee[1,3]

[1]*Department of Physics, University of Toronto, Ontario M5S 1A7, Canada*

[2]*Department of Physics and Astronomy, McMaster University, Hamilton, Ontario L8S 4M1, Canada*

[3]*Canadian Institute for Advanced Research, CIFAR Program in Quantum Materials, Toronto, ON M5G 1M1, Canada*

(Dated: May 2, 2019)

Supplementary Note 1: Honeycomb Strip and Hidden Points of SU(2) Symmetry

In this work, we studied the $K\Gamma$ model as defined by the Hamiltonian of Eq. (1) in the main text on a two-leg honeycomb strip using numerical exact diagonalization (ED) and density matrix renormalization group (DMRG). The $K\Gamma$ model defined on this geometry is particularly suited to infer the physics of the 2D honeycomb limit because it supports two points of hidden SU(2) symmetry that are also present in this limit. These points of hidden SU(2) symmetry are revealed by transforming the $K\Gamma$ Hamiltonian via a six-sublattice transformation dubbed $\mathcal{T}_6$[1].

Supplementary Figure 1(a) depicts the two-leg honeycomb geometry, which can be considered as a strip of the honeycomb lattice. Periodic boundary conditions (PBC) on the two-leg cluster are imposed by (i) connecting the outward vertical z-bonds as shown by blue dashed lines in 1(a) and (ii) connecting the ends of the legs, i.e. members of the slashed bonds are identified. The six-sublattice convention is defined according to labels on the sites in Supplementary Figure 1.

We now define $\mathcal{T}_6$ as

$$\begin{array}{llcl} & & \mathcal{T}_6 & \\ \text{sublattice 1:} & (S^x,S^y,S^z) & \to & (\ \widetilde{S}^x,\ \ \widetilde{S}^y,\ \ \widetilde{S}^z), \\ \text{sublattice 2:} & (S^x,S^y,S^z) & \to & (-\widetilde{S}^x,-\widetilde{S}^z,-\widetilde{S}^y), \\ \text{sublattice 3:} & (S^x,S^y,S^z) & \to & (\ \widetilde{S}^y,\ \ \widetilde{S}^z,\ \ \widetilde{S}^x), \\ \text{sublattice 4:} & (S^x,S^y,S^z) & \to & (-\widetilde{S}^y,-\widetilde{S}^x,-\widetilde{S}^z), \\ \text{sublattice 5:} & (S^x,S^y,S^z) & \to & (\ \widetilde{S}^z,\ \ \widetilde{S}^x,\ \ \widetilde{S}^y), \\ \text{sublattice 6:} & (S^x,S^y,S^z) & \to & (-\widetilde{S}^z,-\widetilde{S}^y,-\widetilde{S}^x). \end{array} \tag{1}$$

The above transformation reveals two points of hidden SU(2) symmetry. When $K=\Gamma>0$, we see that $\mathcal{H}\to\widetilde{J}\sum_{\langle j,k\rangle}\widetilde{\mathbf{S}}_j\cdot\widetilde{\mathbf{S}}_k$, with $\widetilde{J}=-K$. Similarly, $K=\Gamma<0$ gives rise to an antiferromagnetic SU(2) point with the same $\widetilde{J}$. We also note that an equivalent $\mathcal{T}_6$ transformation can be defined on the C_3-symmetric (i.e. under cyclic permutations $x\to y\to z\to x$) honeycomb cluster with the sublattices shown in Supplementary Figure 1(b). Periodic boundary conditions on the C_3-symmetric honeycomb cluster are imposed by identifying protruding bonds of the same type on opposite sides of the cluster.

It is important to note that the boundary conditions in these clusters allow for a consistent

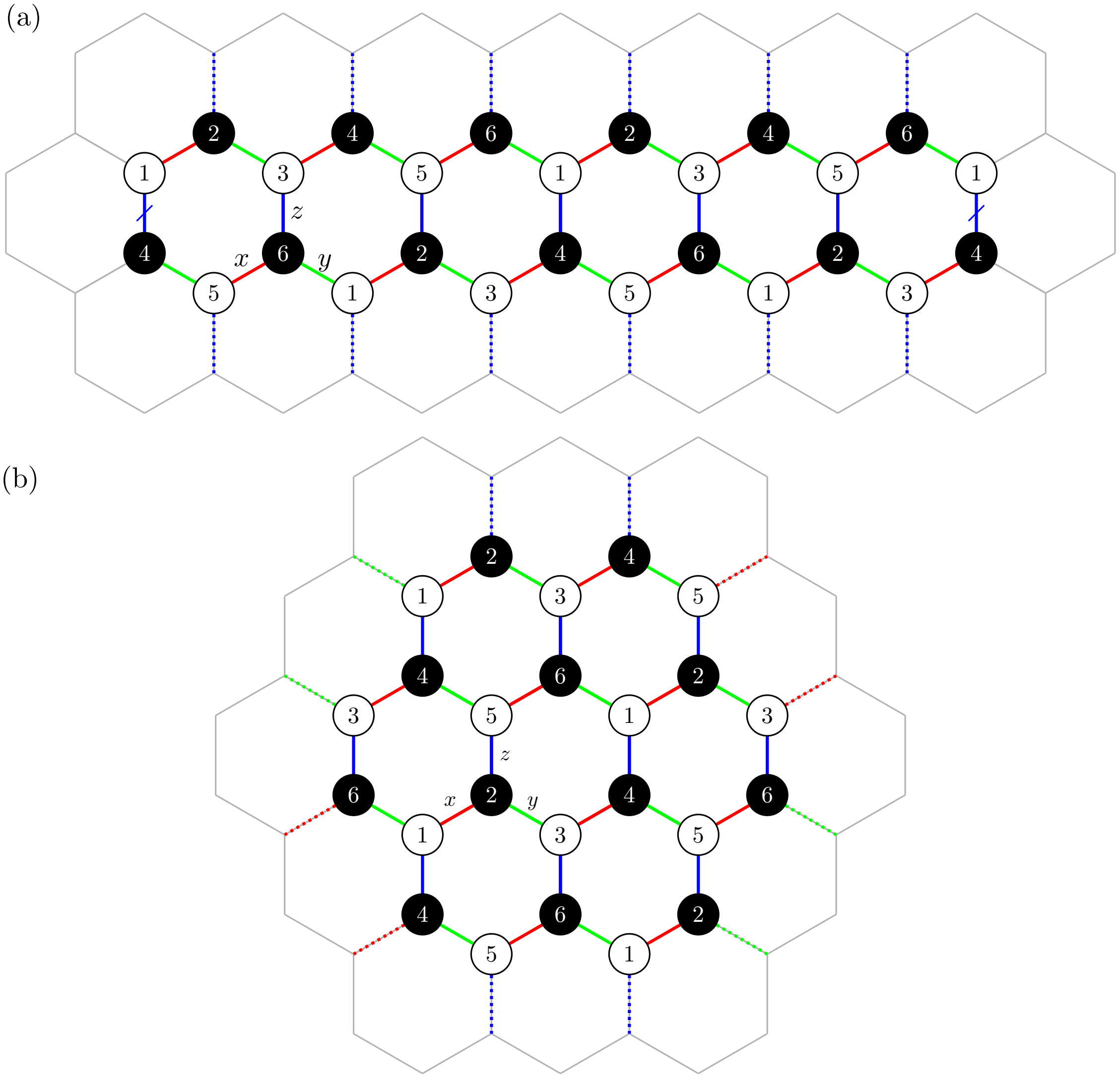

Supplementary Figure 1. **Cluster Geometries** – Clusters with $N = 24$ sites used in the ED calculations. The bond types $\gamma = x, y, z$ in the Hamiltonian are shown as red, green, and blue bonds, respectively, and labelled around one site. The six-sublattice convention used for $\mathcal{T}_6$ is indicated by the numbers on each site. (a) Two-leg honeycomb geometry depicted as a strip of the honeycomb lattice. Periodic boundary conditions are imposed by (i) connecting protruding z bonds vertically depicted by blue dashed lines, and (ii) identifying the slashed bonds at the left and right ends of the legs. (b) C_3-symmetric honeycomb cluster, where periodic boundary conditions are imposed by identifying protruding bonds on opposite ends of the cluster.

definition of the $\mathcal{T}_6$ transformation, and thus support points of hidden SU(2) symmetry present in the infinite honeycomb lattice. For this reason, the two-leg and C_3-symmetric honeycomb clusters described here are suitable choices.

Supplementary Note 2: Role of Γ'

In addition to components of the spin-spin correlators, the spin structure factor $S(\mathbf{q})$ can be used to distinguish the KΓSL from ZZ magnetic order and the KSL. In Supplementary Figure 2 we show $S(\mathbf{q})$ at $\mathbf{q} = \mathbf{\Gamma}$ and $\mathbf{M}$ as a function of Γ/K at $h = 0$ calculated in the C_3-symmetric honeycomb cluster with ED for different values of Γ'. As a negative Γ' is introduced, $\mathbf{M}$-point correlations quickly dominate over $\mathbf{q} = \mathbf{\Gamma}$ outside of the KSL as shown in 2(a-c). The emergence of peaks in $S(\mathbf{q})$ at $\mathbf{q} = \mathbf{M}$ indicate the onset of ZZ magnetic order due to Γ', and sharpen as the magnitude of Γ' increases[2]. The KSL phase is also seen to shrink, pushing it towards the pure K limit indicated by $\chi_{\Gamma/K}$ in 2(d-f). Beyond a critical value of $\Gamma'_{\mathrm{c}} \simeq -0.1$, the KSL phase is destroyed entirely in favour of ZZ order. Inclusion of subdominant FM nearest-neighbour Heisenberg (J) and dominant AFM third neighbour Heisenberg (J_3) also tends to promote ZZ magnetic order alongside the FM Γ'. If the combined effect of Γ'-J-J_3 is too large, the KSL phase may be completely destroyed. Observation of the thermal Hall effect in α-$RuCl_3$ suggests that the combined effect of these terms is small, but quantifying this is left as future work.

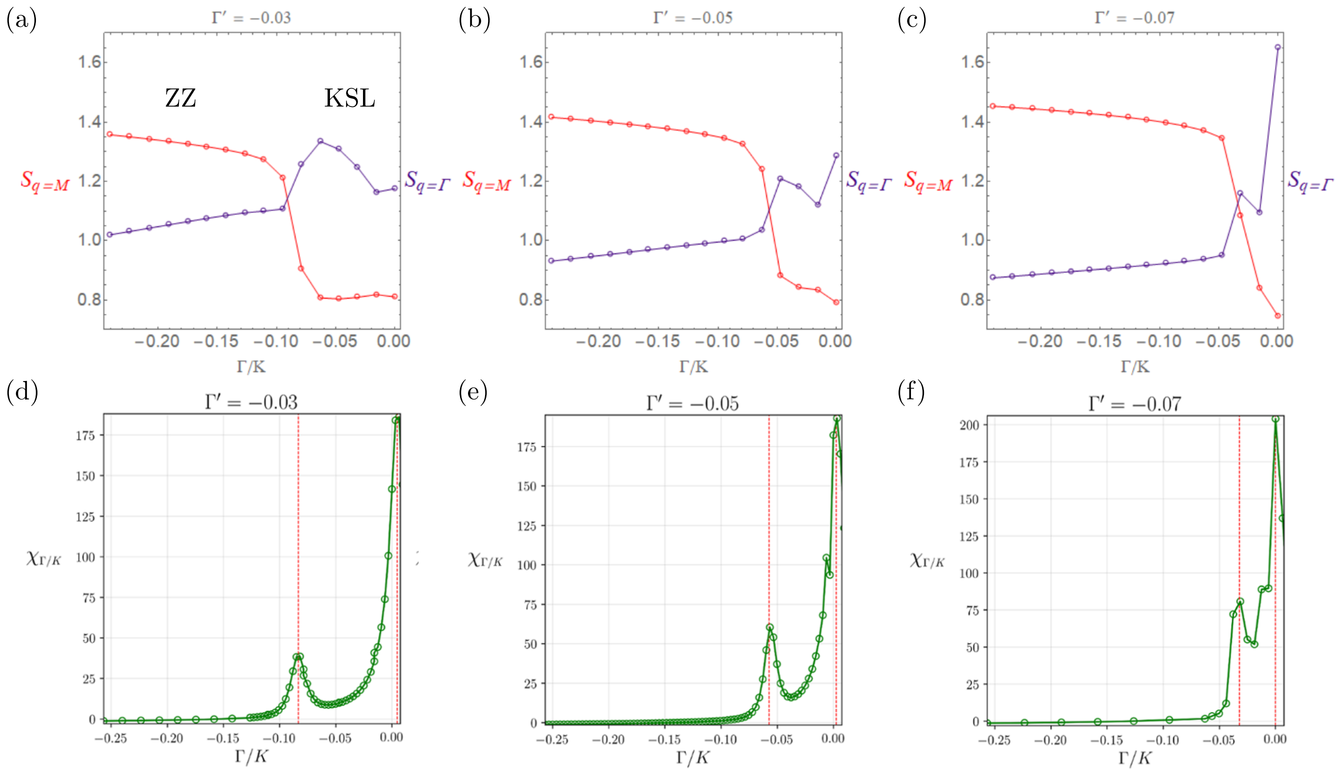

Supplementary Figure 2. **Role of $\mathbf{\Gamma'}$** – Zero-field values of (a-c) the spin structure factor $S(\mathbf{q})$ at $\mathbf{q} = \mathbf{M}, \mathbf{\Gamma}$ and (d-f) $\chi_{\Gamma/K}$ as a function of Γ/K calculated with ED on the C_3-symmetric honeycomb cluster for different values of Γ'. With a finite FM Γ', the KΓSL is replaced by ZZ magnetic order as discussed in the main text. ZZ order is characterized by dominant **M**-point correlations which grow with the magnitude of Γ', while $\mathbf{\Gamma}$-point correlations are favoured in the KSL. At the same time, peaks in $\chi_{\Gamma/K}$ show that the KSL phase space shrinks until it disappears entirely at $\Gamma'_c \simeq -0.1$.

Supplementary Note 3: Plaquette Expectation Values

In the pure Kitaev limit the W_p operators are conserved quantities, and the ground state is characterized by $W_p = +1$ on all plaquettes. Although this is no longer true with finite h, Γ, or Γ', $\langle W_p \rangle$ is still a quantity which can distinguish the KSL from the nearby ZZ order. We therefore seek an analytic expression for $\langle W_p \rangle$ in the relevant magnetically ordered states. First, we start with a FM arrangement of spins, where the moment on each site points along the [111] direction with state $|\uparrow_{111}\rangle$. The moments are then uniformly rotated through polar angles (θ, φ), where θ is defined from the [111] axis and φ from the $[11\overline{2}]$ axis. With $\varphi = 0$, θ corresponds to the tilting angle of the field in the $\mathbf{\hat{a}\hat{c}^*}$ plane considered in the main text.

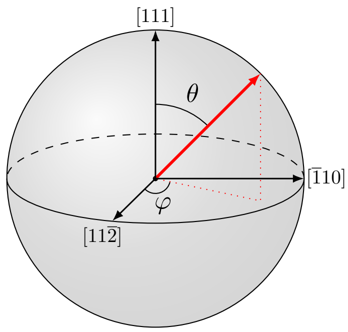

$$|\Psi_{\mathrm{FM}}(\theta,\varphi)\rangle = \prod_{j=1}^{N} \mathcal{R}^{(j)}_{111}(\varphi)\mathcal{R}^{(j)}_{\bar{1}10}(\theta)\,|{\uparrow_{111}}\rangle_j \tag{2a}$$

$$\mathcal{R}^{(j)}_{111}(\varphi) = \exp\left(-i\frac{\varphi}{2}\frac{1}{\sqrt{3}}(\sigma^x_j+\sigma^y_j+\sigma^z_j)\right) \tag{2b}$$

$$\mathcal{R}^{(j)}_{\bar{1}10}(\theta) = \exp\left(-i\frac{\theta}{2}\frac{1}{\sqrt{2}}(-\sigma^x_j+\sigma^y_j)\right). \tag{2c}$$

Expectation value of the plaquette operator in this state is found to be

$$\langle W_p\rangle_{\mathrm{FM}}(\theta,\varphi) = \left(\frac{3\cos(\theta)+5\cos(3\theta)-4\sqrt{2}\sin^3(\theta)\cos(3\varphi)}{24\sqrt{3}}\right)^2. \tag{3}$$

This vanishes unless the moment direction involves all three spin components, and is maximized along the [111] ($\theta=0$) and symmetry-related directions. Similarly, a collinear AFM product state ansatz for the zig-zag magnetic order yields the same plaquette expectation but with an overall negative sign. While the expression for $\langle W_p\rangle_{\mathrm{FM}}$ becomes exact in the limit of high fields, the ZZ counterpart is not exact due to the product state ansatz. However, the overall sign of $\langle W_p\rangle$ is correctly captured within this approximation.

Supplementary Note 4: Effect of a Magnetic Field Perpendicular to the $\mathbf{\hat{a}\hat{c}^*}$ Plane

For completeness, we have also investigated the K-Γ-Γ'-h model under a magnetic field in the $[1\bar{1}0]$ direction perpendicular to the $\mathbf{\hat{a}\hat{c}^*}$ plane. Components of the spin-spin correlators in the Γ/K-h plane are shown in Supplementary Figure 3 as calculated with DMRG in the two-leg honeycomb cluster for $\Gamma' = -0.1$. Phase boundaries determined by peaks in the magnetic and Γ/K susceptibilities are shown as red lines. The most striking difference from other field directions is the immediate instability of the KSL. This contrasts the 24-site honeycomb cluster studied with ED, where we have found that the KSL region for a $[1\bar{1}0]$ field is similar to that of the $[11\bar{2}]$ direction. These observations are consistent with a mirror symmetry of the model which prevents a finite thermal Hall conductivity[3], but the phase boundary found with DMRG is an artifact of the strip geometry. As with the other directions considered, the ZZ magnetic order extends to finite field at larger $\Gamma/|K|$.

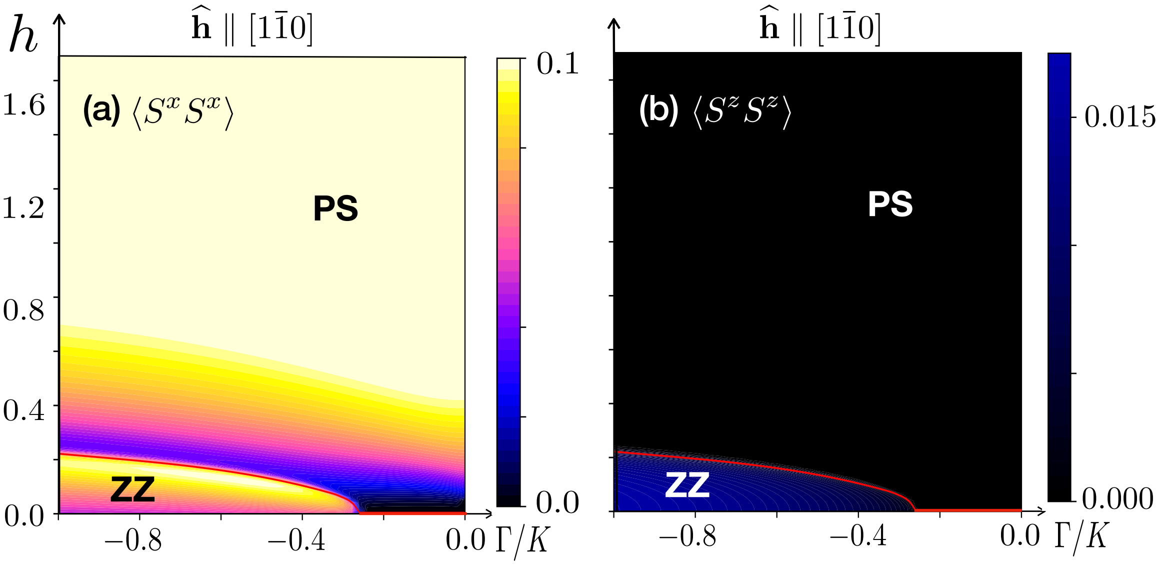

Supplementary Figure 3. **Magnetic Field in the [1$\bar{1}$0] Direction** – (a) $\langle S^x_j S^x_k \rangle$ and (b) $\langle S^z_j S^z_k \rangle$ evaluated at $k - j = 50$ is shown as a function of Γ/K and [1$\bar{1}$0] field strength for $\Gamma' = -0.1$. These are obtained with DMRG in the two-leg honeycomb cluster with $N = 200$ and OBC. Smooth curves fitted to singular features in either χ_h or $\chi_{\Gamma/K}$ are drawn as red lines. Unlike magnetic fields in the $\hat{\mathbf{a}}\hat{\mathbf{c}}^*$ plane, the KSL phase is immediately unstable to a magnetic field in the [1$\bar{1}$0] direction for the two-leg honeycomb strip, indicated by the phase boundary in red at $h = 0$. ZZ magnetic order extends to finite field, until it is destroyed in favour of the polarized state. The $\langle S^x S^x \rangle$ (= $\langle S^y S^y \rangle$) correlations tend to a finite value in high fields, while $\langle S^z S^z \rangle$ correlations tend to zero because the field lies in the $\hat{\mathbf{x}}\hat{\mathbf{y}}$ plane.

---

[1] Ji ří Chaloupka and Giniyat Khaliullin, "Hidden symmetries of the extended Kitaev-Heisenberg model: Implications for the honeycomb-lattice iridates A_2IrO_3," Phys. Rev. B **92**, 024413 (2015).

[2] Jeffrey G. Rau and Hae-Young Kee, "Trigonal distortion in the honeycomb iridates: Proximity of zigzag and spiral phases in Na_2IrO_3," preprint at http://arXiv.org/abs/1408.4811 (2014).

[3] Liujun Zou and Yin-Chen He, "Field-induced neutral Fermi surface and QCD_3-Chern-Simons quantum criticalities in Kitaev materials," preprint at http://arXiv.org/abs/1809.09091 (2018).
